# Immune-Inhibitory Gene Expression is Positively Correlated with Overall Immune Activity and Predicts Increased Survival Probability of Cervical and Head and Neck Cancer Patients

**DOI:** 10.3389/fmolb.2021.622643

**Published:** 2021-03-23

**Authors:** Megha Budhwani, Gavin Turrell, Meihua Yu, Ian H. Frazer, Ahmed M. Mehdi, Janin Chandra

**Affiliations:** The University of Queensland Diamantina Institute, The University of Queensland, Woolloongabba, QLD, Australia

**Keywords:** cervical cancer, head and neck cancer, inflamed tumours, immune inhibition, immune checkpoints, prognosis

## Abstract

**Background:** Limited immunotherapy options are approved for the treatment of cervical cancer and only 10–25% of patients respond effectively to checkpoint inhibition monotherapy. To aid the development of novel therapeutic immune targets, we aimed to explore survival-associated immune biomarkers and co-expressed immune networks in cervical cancer.

**Methods:** Using The Cancer Genome Atlas (TCGA) Cervical Squamous Cell Carcinoma (CESC) data (*n* = 304), we performed weighted gene co-expression network analysis (WGCNA), and determined which co-expressed immune-related genes and networks are associated with survival probability in CESC patients under conventional therapy. A “Pan-Immune Score” and “Immune Suppression Score” was generated based on expression of survival-associated co-expressed immune networks and immune suppressive genes, which were subsequently tested for association with survival probablity using the TCGA Head Neck Squamous Cell Carcinoma (HNSCC) data (*n* = 528), representing a second SCC cancer type.

**Results:** In CESC, WGCNA identified a co-expression module enriched in immune response related genes, including 462 genes where high expression was associated with increased survival probability, and enriched for genes associated with T cell receptor, cytokine and chemokine signaling. However, a high level of expression of 43 of the genes in this module was associated with decreased survival probability but were not enriched in particular pathways. Separately, we identified 20 genes associated with immune suppression including inhibitory immune checkpoint and regulatory T cell-related genes, where high expression was associated with increased survival probability. Expression of these 20 immune suppressive genes (represented as “Immune Suppression Score”) was highly correlated with expression of overall survival-associated immune genes (represented as “Pan-Immune Score”). However, high expression of seven immune suppression genes, including TWEAK-R, CD73, IL1 family and TGFb family genes, was significantly associated with decreased survival probability. Both scores also significantly associated with survival probability in HNSCC, and correlated with the previously established “Immunophenoscore.”

**Conclusion:** CESC and HNSCC tumors expressing genes predictive of T cell infiltrates (hot tumors) have a better prognosis, despite simultaneous expression of many immune inhibitory genes, than tumors lacking expression of genes associated with T cell infiltrates (cold tumors) whether or not these tumor express immune inhibitory genes.

## Introduction

Cervical squamous cell carcinoma (CESC) resulted in an estimated 311,000 deaths and 570,000 new cases in 2018 worldwide, representing a major health burden (Paul et al., 2019). Virtually all CESC cases arise from infection by oncogenic strains of the human papillomavirus (HPV), which is also the causative agent of other anogenital and oropharyngeal cancers (Zhou et al., 2019), such as a proportion of head and neck squamous cell carcinoma (HNSCC). In contrast, HPV-negative HNSCCs are primarily caused by heavy smoking and/or drinking (Johnson et al., 2020). While a preventive HPV vaccine exists, CESC and HPV-positive HNSCC-associated deaths continue to occur in low and middle-income countries, where the vaccine remains unaffordable. Regular cervical screening facilitates early detection, but 5-years survival of women with stage III CESC lies below 40% (Otter et al., 2019). Meanwhile, the cases of HNSCC rise continually with 450,000 deaths in 2018 (Johnson et al., 2020). Therefore, CESC and HNSCC will likely remain significant global health issues.

The immune system plays an important role in the prevention and recognition of cancer. Different patients with the same cancer type have widely differenting tumor immune infiltrates. Tumors with significant tumor infiltrating lymphocytes (TILs) are characterized as “hot” and those with limited infiltrates are referred to as “cold” (Galon and Bruni, 2019). For many cancers, including colorectal cancer (Narayanan et al., 2018) and cervical cancer (De Vos van Steenwijk et al., 2013), hot tumors are associated with better survival. A “cold” tumor may reflect lack of tumor specific antigens, defective antigen presentation, lack of T cell activation or deficiencies in migration of immune cells to the tumor site (Bonaventura et al., 2019). This simplified categorization of “hot” and “cold” tumors however doesn’t take the mechanisms of active immune suppression which are commonly observed within tumors into consideration. Particularly, inhibitory immune molecules displayed by cancer and/or immune cells can contribute to prevention of tumor cell-targeted effector T cell responses. The heterogeneic nature of cancers including CESC thus requires methods for tumor stratification for prognostic purposes beyond the classical Tumor/Nodes/Metastasis (TNM) staging system, including the consensus Immunoscore (Pagès et al., 2018; Angell et al., 2020). Identification of prognostic immune-related gene signatures and the “Immunophenoscore” facilitated retrospective patient stratification in CESC (Charoentong et al., 2017; Thorsson et al., 2018; Yang et al., 2019; Nie et al., 2020; Qi et al., 2020) and will potentially guide immunotherapy selection to increase survival outcomes. However, identification of co-expressed immune networks will help to identify optimal targets for therapy. To the best of our knowledge, there is no report comprehensively evaluating the co-expression of immune-related genes and networks in CESC that are significantly associated with survival probability.

Herein, we utilized weighted gene co-expression network analysis (WGCNA) to explore the central immune networks associated with probability of survival for patients receiving conventional therapy. Using the TCGA-CESC dataset, we identified a novel co-expression Immune Module, comprising immune genes for which high expression was associated with increased survival probability. The survival-associated co-expressed immune gene landscape clustered in pathways of T cell activity and infiltration promoting chemokine and cytokine networks, and contained many genes associated with immune-suppression. High expression of 20 inhibitory immune genes was paradoxically associated with increased survival probability, of which 16 were co-expressed in the WGCNA Immune Module, while only high expression of seven inhibitory genes was associated with decreased survival probability. Based on the identification of survival-associated stimulating and inhibitory immune genes in CESC associating with increased survival probability, we established an overall “Pan-Immune Score” and an “Immune Suppression Score” respectively, which were highly positively correlated in both primary CESC and HNSCC. These scores were also highly positively correlated with the previously proposed “Immunophenoscore” (Charoentong et al., 2017). Overall, this study shows that phenotypic stratification of CESC and HNSCC tumors based on co-expressed immune networks or expression of specific immune suppressive genes unexpectedly reveals a hierarchy of survival probability, in which “hot” tumors with significant T cell infiltrates that co-express present mechanisms of specific immune suppression have a better prognosis than “cold” tumors lacking T cell infiltrates.

## Methods

### Data Collection and Filtering

The TCGA-CESC clinical data was obtained from the GDC legacy database using the TCGAbiolinks R package (https://bioconductor.org/packages/release/bioc/html/TCGAbiolinks.html). The legacy mRNA TCGA-CESC expression data (Illumina HiSeqV2) was manually downloaded from XenaBrowser (https://tcga.xenahubs.net/download/TCGA.CESC.sampleMap/HiSeqV2.gz). Both clinical and expression data was filtered to include “Primary Tumor” sample types only, using the unique descriptive barcode assigned to each patient, 304 patients remained after applying this filter. The clinicopathological data of this cohort has been described previously (Song et al., 2019). We also downloaded the normalized gene expression and clinical data of TCGA-HNSCC from XenaBrowser (https://tcga.xenahubs.net/download/TCGA.HNSC.sampleMap/HiSeqV2_PANCAN.gz). Similar to the cervical cancer data, TCGA-HNSCC was filtered to include “Primary Tumor” sample types only.

### Weighted Gene Co-expression Network Analysis

We developed a weighted co-expression network of the TCGA-CESC expression data using the WGCNA package in R (Langfelder and Horvath, 2008). The co-expression was measured between the genes in the TCGA-CESC data by using a Pearson correlation function. The adjacency matrix was constructed by raising the co-expression similarity power to β = 6. From the adjacency matrix, we built the topological overlap matrix (TOM) to consider topological similarity and a corresponding dissimilarity matrix (1-TOM) to form clusters. The hclust function was used to perform hierarchical clustering using the dissimilarity matrix. The flashClust package was used to determine the outliers (Langfelder and Horvath, 2012). We used the dynamic tree cutting algorithm with parameters deep split = 2, minimum gene modules = 20, cut height = 0.6 to detect highly distinct gene modules. Module Eigengene expressions (ME) represented by the first principal component (PC1) were calculated to determine the expression of each module. Clinical data was correlated with Eigengene expression of modules and gene significance (GS) was determined. A quantitative method of module membership was determined by correlating ME of modules with gene expression of modules. Colors were randomly assigned to modules. To provide gene ontology (GO) enrichment analysis for modules, the top three gene ontology processes were determined for each module using enrichGO function in the clusterProfiler package (Yu et al., 2012). To identify immune-related modules, GO terms containing the text “immune” were searched. In this research only one module (palevioletred1) was identified as Immune Module.

### Kaplan Meier Survival Analysis

KMSA was performed using customized R scripts (http://www.r-project.org/). Patients’ gene expression, clinical and scoring data was matched using the assigned patient barcodes. For each gene expression or score, patients were assigned to two groups; Upper Quartile (UQ) and Lower Quartile (LQ) based on whether their gene expression or score values were in the bottom 25% or the top 25% of gene expression or score. KMSA was performed with the R package Survminer (https://cran.r-project.org/web/packages/survminer/index.html) using default arguments and Rho = 0, and the R package *Survival* (https://cran.r-project.org/web/packages/survival/index.html) using default settings to extract a log rank test *p*-value for overall survival of up to 1825 days (5 years). *p*-values of significant associations were −log10 transformed for visualization.

### Pathway Analysis

Significant survival-associated genes were sorted into groups of high and low expression in CESC patients with increased survival outcomes. The resulting gene lists were separately analyzed using Enrichr (http://amp.pharm.mssm.edu/Enrichr/) followed by mapping to human KEGG 2019 Human pathways and GO Biological Process 2018 ontologies.

### Protein-Protein-Interaction Networks

PPIs were predicted using the Search Tool for the Retrieval of Interacting Genes (STRING) (https://string-db.org/) based on a threshold of interaction score >0.9. Hub genes were identified as genes with the highest degree of connectivity for the top enriched pathways.

### Developing the “Pan-Immune Score” and “Immune Suppression Score”

To establish an “Immune Suppression Score,” we created an immune suppression gene list consisting of 50 genes and including checkpoint inhibitory genes, Treg-associated genes and immune-suppressive cytokines, which was tested for survival association using KMSA. The “Immune Suppression Score” was created based on 20 of these genes for which high expression was associated with increased survival probability (Supplementary File S1). To establish a “Pan-Immune Score,” we used the 462 immune genes identified from the WGCNA palevioletred1 Immune Module for which high expression was associated with increased survival probability, excluding immune suppression genes which were part of the “Immune Suppression Score” to ensure both scores are independent (Supplementary File S1).

The establishment of the “Pan-Immune Score” and “Immune Suppression Score” was performed using a method developed by Foroutan and colleagues (Foroutan et al., 2018). Specifically, each sample in the TCGA-CESC and TCGA-HNSCC expression data was ranked by increasing transcript abundance using the rankgenes function in the singscore package (Foroutan et al., 2018). The mean rank was calculated and normalized against the minimum and maximum value of the rank in all samples in the gene expression datasets. A high “Pan-Immune Score” and “Immune Suppression Score” represents that a particular sample are in concordant with the immune and immune suppression gene pattern. Individual scores for each TCGA CESC and HNSCC sample are reported in Supplementary File S1. We compared 5-years survival probability of the upper and lower quartiles of the “Pan-Immune Score” and “Immune Suppression Score” using KMSA.

### “Immunophenoscore” Analysis

“Immunophenoscore” data (IPS1-4) for CESC and HNSCC was retrieved from www.tcia.at and are reported in Supplementary File S1. Briefly, IPS1 is calculated based on gene or metagene expression in four catagories (effector cells, suppressive cells, MHC-related molecules, immunomodulators) (Charoentong et al., 2017). An average z-score based on gene expression in each category was calculated and, and was subsequently positively weighted for catagories “effector cells,” “MHC-related molecules” and for stimulatory “immunomodulator” genes, and negatively weighted for the catagory “suppressive cells” and for inhibitory “immunomodulator” genes. Three additional IPS scores (IPS2-4) were calculated with the aim to facilitate predicting response to checkpoint treatment, where the inhibitory “immunomodulator” gene to be blocked was positively weighted instead (IPS2 positivly weighted expression of CTLA-4; IPS3 positively weighted expression of PD1/PDL1/PDL2; IPS4 positivly weighted expression of CTLA-4/PD1/PDL1/PDL2).

### Correlation Analysis

Pearson R correlation analysis (PRCA) was used to determine correlation between WGCNA modules with gene significance, between “Pan-Immune Score,” “Immune Suppression Score” and the “Immunophenoscore” (IPS1-4). Spearman correlation analysis was used to determine correlation between survival-associated immune genes and FIGO stage.

### KEGG Immune Pathway Gene Selection

Immune pathway gene lists were generated using KEGG (http://www.genome.jp/kegg/). KEGG pathways analyzed were: NF-kappa B signaling pathway (hsa04064), Antigen Processing and Presentation (hsa04612), B cell Receptor Signaling Pathway (hsa04662), C-Type lectin receptor signaling pathway (hsa04625), Cell Adhesion (hsa04514), Chemokine Signaling (hsa04062), Complement and Coagulation Cascades (hsa04610), Jak-STAT signaling pathway (hsa04630), Cytosolic DNA-sensing pathway (hsa04623), Extracellular Matrix (hsa04512), Fc Epsilon RI signaling pathway (hsa04664), Fc Gamma R-mediated phagocytosis (hsa04666), Hematopoietic Cell Lineage (hsa04640), IL-17 Signaling Pathway (hsa04657), Intestinal Immune Network for IgA Production (hsa04672), Leukocyte Transendothelial Migration (hsa04670), Natural Killer Cell Mediated Cytotoxicity (hsa04650), NOD-like receptor signaling Pathway (hsa04621), Platelet Activation (hsa04611), RIG-I-like Receptor Signaling Pathway (has04622), T Cell Receptor Signaling (hsa04660), Th1 and Th2 Differentiation (hsa04658), Th17 Differentiation (hsa04659), Toll-like Receptor Signaling (hsa04620), Toll and Imd Signaling Pathway (hsa04624). Genes were analyzed for survival association using KMSA.

## Results

### Co-Expression of Immune Genes Is Associated With Increased CESC Survival

**FIGURE 1:**
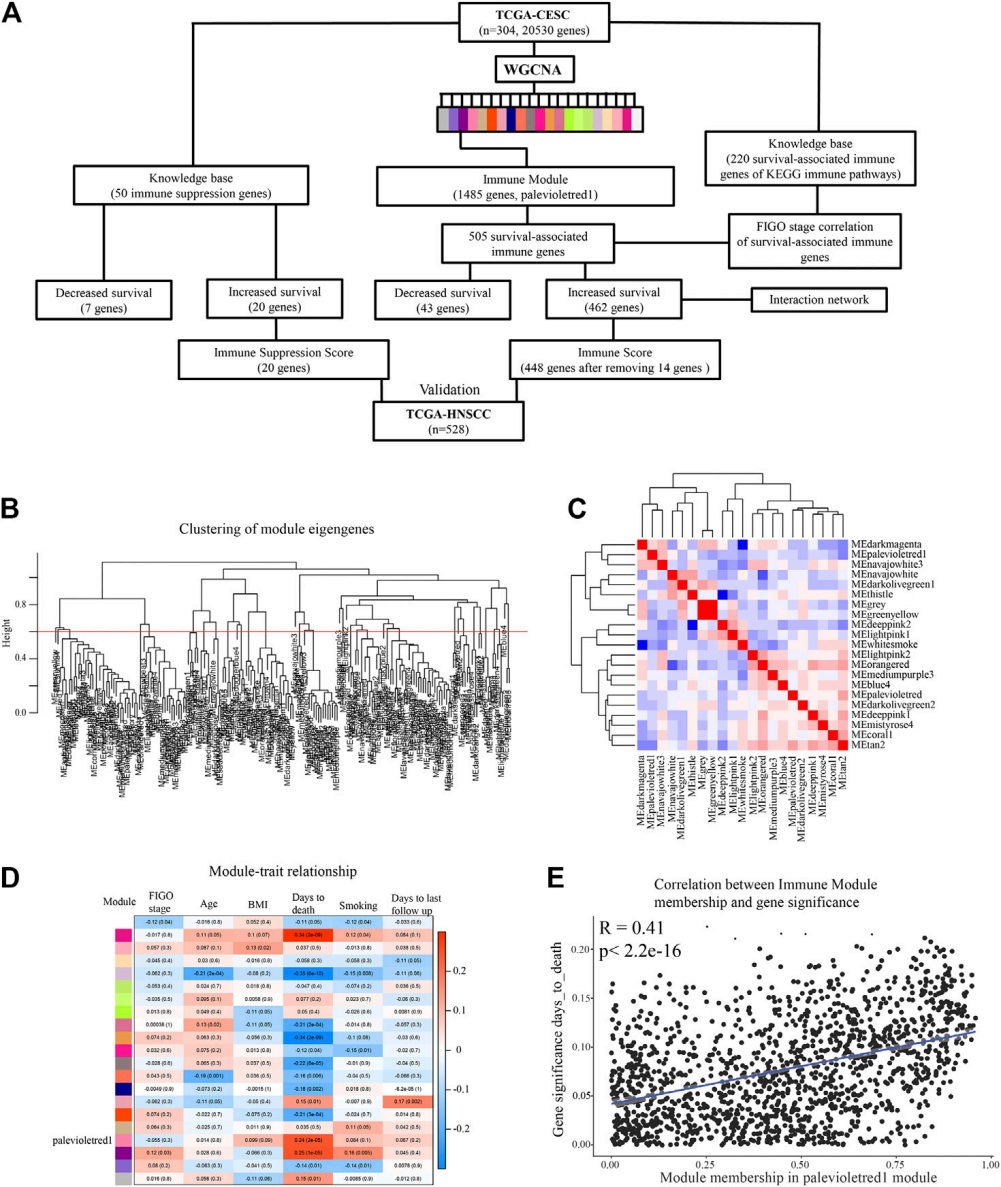
Idenditifaction of a co-expression Immune Module which correlates with CESC survival probability. **(A)** Flow chart describing analysis pipeline and summary of results. **(B)** Weighted gene co-expression network analysis (WGCNA) was used to identify survival-associated co-expressed immune networks in the TCGA CESC data. Clustering of module eigengenes with a threshold of 0.6 resulted in 21 modules. **(C)** Correlation was performed between eigengene expressions of each module with each other to represent association of modules with each other. **(D)** Correlation analysis of modules eigengene expression with clinical features. **(E)** Correlation analysis comparing the palevioletred1 Immune Module to clinical feature days_to_death.

We sought to identify co-expressed immune genes and networks predictive of probability of survival for patients with CESC. Weighted gene co-expression network analysis (WGCNA) across all 20,530 genes of TCGA data for 304 patients with CESC using a co-expression threshold of 0.6 resulted in 21 co-expression modules (Figures 1A,B), with generally weak intermodule correlation, with the exception of the gray and greenyellow module (Figure 1C), validating the chosen co-expression threshold as appropriate for module stratification. Gene Ontology (GO) analysis showed that the palevioletred1 module was enriched in immune receptor and cytokine receptor activity (Table 1), while correlation analysis identified that this module was significantly associated with days to death for the subjects in the TCGA cohort, but associated with no other recorded clinical features (Figures 1D,E).

**TABLE 1:** Enriched GO biological processes for each WGCNA module. Only top three are shown. NA: not applicable, no GO ID was identified.

Modules	No. of genes	GO IDs	GO description
palevioletred1	1,485	GO: 0140375, GO: 0004896, GO: 0030246	Immune receptor activity, cytokine receptor activity, carbohydrate binding
Whitesmoke	2,643	GO: 0046873, GO: 0015370, GO: 0015077	Metal ion transmembrane transporter activity, solute: sodium symporter activity, monovalent inorganic cation transmembrane transporter activity
Gray	786	NA	NA
Darkmagenta	1892	GO: 0030280, GO: 0098631, GO: 0098632	Structural constituent of skin epidermis, cell adhesion mediator activity, cell-cell adhesion mediator activity
Greenyellow	3,361	GO: 0019787, GO: 0004842, GO: 0016887	Ubiquitin-like protein transferase activity, ubiquitin-protein transferase activity, ATPase activity
deeppink2	740	GO: 0018455	Alcohol dehydrogenase [NAD(P)+] activity
Navajowhite	1,184	GO: 0005201, GO: 0005539, GO: 0005518	Extracellular matrix structural constituent, glycosaminoglycan binding, collagen binding
Orangered	5,881	GO: 0004984, GO: 0003735, GO: 0005549	Olfactory receptor activity, structural constituent of ribosome, odorant binding
lightpink2	61	NA	NA
lightpink1	260	NA	NA
Thistle	614	GO: 0030545, GO: 0070851, GO: 0048018	Receptor regulator activity, growth factor receptor binding, receptor ligand activity
mistyrose4	229	GO: 0008242, GO: 0140,097	omega peptidase activity, catalytic activity, acting on DNA
mediumpurple3	130	NA	NA
tan2	517	GO: 0004364, GO: 0098960, GO: 0030594	Glutathione transferase activity, postsynaptic neurotransmitter receptor activity, neurotransmitter receptor activity
coral1	130	NA	NA
Palevioletred	46	NA	NA
darkolivegreen1	249	NA	NA
deeppink1	81	GO: 0070325, GO: 0008201, GO: 0048018	Lipoprotein particle receptor binding, heparin binding, receptor ligand activity
darkolivegreen2	133	NA	NA
navajowhite3	43	GO: 0030247, GO: 0030246	Polysaccharide binding, carbohydrate binding
blue4	65	NA	NA

**FIGURE 2:**
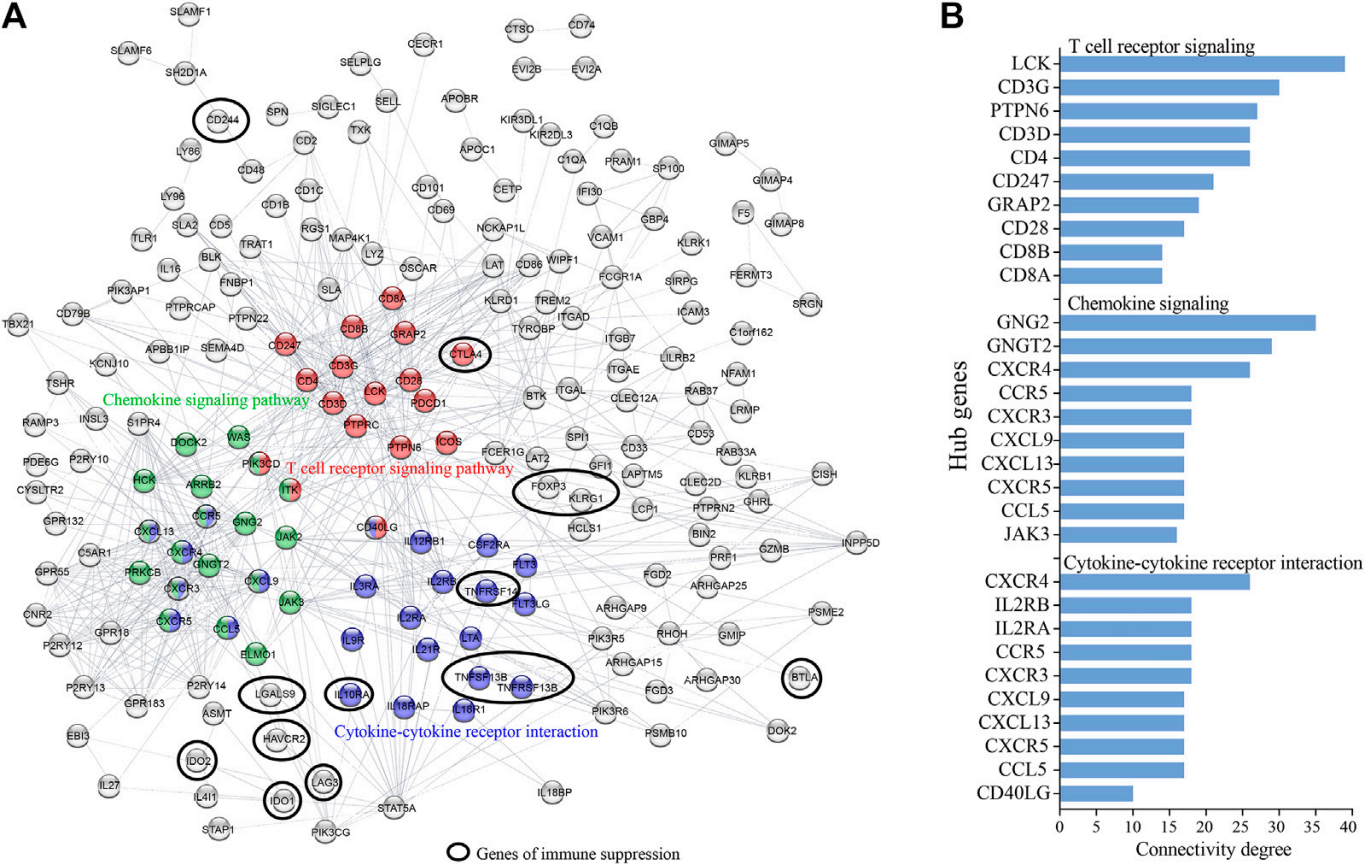
Protein-protein interaction (PPI) network identifies hub network genes promoting increased CESC survival probability. Co-expressed survival-associated immune genes of the WGCNA palevioletred1 Immune Module were analyzed for PPI. **(A)** Shown is the network of 462 highly expressed genes in patients of increased survival probability (excluding non-connected genes). **(B)** Hub genes were identified as genes with highest connectivity in a particular KEGG pathway.

The palevioletred1 Immune Module comprised a total of 1,485 genes (Figure 1A). As this module showed significant correlation with days to death, and the strongest GO enrichment in immune related processes, we assessed each of the genes of this module for significant association with 5-years survival probability using Kaplan Maier Survival analysis (KMSA) and comparing top and bottom quartiles of gene expression. From this module, we identified 462 genes where high level expression was significantly associated with increased 5-years survival probability and 43 genes where high level expression was significantly associated with decreased 5-years survival probability (Figure 1A; Supplementary File S1). A majority of the 462 genes where high level expression was associated with increased 5-years survival were linked to immune processes according to GO and KEGG pathway analysis (Table 2), whereas the 43 co-expressed genes where high level expression was significantly associated with decreased 5-years survival probability were not particularly representative of any GO or KEGG pathway (data not shown). To determine genes with increased connectivity from amongst the 462 genes associated with increased 5-years survival probability, we built a protein-protein interaction (PPI) network. These genes organized into three major hubs overlapping with the T cell receptor signaling, chemokine signaling and cytokine-cytokine receptor interaction KEGG pathways (Figure 2A). Central genes of these hubs were LCK, CD3G, PTPN6, CD3D, and CD4 in the T cell receptor signaling pathway; GNG2, GNGT2, CXCR4, CCR5, and CXCR3 in chemokine signaling pathway; and CXCR4, IL2RB, IL2RA, CCR5, and CXCR3 in the cytokine-cytokine receptor interaction pathway (Figure 2B). CXCL9-CXCR3 interaction and CCL5 are crucial for T cell function and infiltration into tumors (Duan et al., 2020), and higher level of expression of these genes might result in improved anti-tumor immunity in patients with better survival outcomes. In contrast, the 43 highly co-expressed genes in patients with decreased survival outcomes arranged themselves randomly without evident connection hubs (data not shown), indicating that there is no specific immune network which is highly expressed in patients with decreased survival probability.

**TABLE 2:** Enriched GO biological processes and KEGG pathways of 462 highly expressed genes in subjects with increased 5-years survival probability.

**2018 GO biological process**	**−log10 *p*-value**
Regulation of immune response (GO: 0050776)	18.7058
T Cell activation (GO: 0042110)	14.1283
Positive regulation of lymphocyte proliferation (GO: 0050671)	9.1974
Cytokine-mediated signaling pathway (GO: 0019221)	9.2134
Lymphocyte differentiation (GO: 0030098)	9.2056
T Cell differentiation (GO: 0030217)	8.9109
Cellular defense response (GO: 0006968)	8.9468
Regulation of T cell activation (GO: 0050863)	8.1090
Positive regulation of T cell activation (GO: 0050870)	7.7301
Inflammatory response (GO: 0006954)	7.7082
Antigen receptor-mediated signaling pathway (GO: 0050851)	7.5528
Regulation of T cell proliferation (GO: 0042129)	7.3419
Cellular response to cytokine stimulus (GO: 0071345)	7.2260
B Cell activation (GO: 0042113)	6.4053
Positive regulation of T cell proliferation (GO: 0042102)	6.4351
Enzyme linked receptor protein signaling pathway (GO: 0007167)	6.1125
Positive regulation of interferon-gamma production (GO: 0032729)	6.1040
Peptidyl-tyrosine autophosphorylation (GO: 0038083)	5.9003
T Cell receptor signaling pathway (GO: 0050852)	5.6791
Peptidyl-tyrosine phosphorylation (GO: 0018108)	5.1200
Positive regulation of interferon-gamma secretion (GO: 1902715)	5.0358
Regulation of B cell proliferation (GO: 0030888)	5.0545
Negative regulation of T cell activation (GO: 0050868)	4.9182
Negative regulation of lymphocyte activation (GO: 0051250)	4.5489
Regulation of defense response to virus by virus (GO: 0050690)	4.4914
Regulation of lymphocyte activation (GO:0051249)	4.3648
Positive regulation of B cell proliferation (GO:0030890)	4.2676
Regulation of natural killer cell mediated cytotoxicity (GO:0042269)	4.1124
Regulation of interferon-gamma secretion (GO:1902713)	3.8788
B Cell receptor signaling pathway (GO:0050853)	3.8575
Dendritic cell chemotaxis (GO:0002407)	3.7978
Positive regulation of antigen receptor-mediated signaling pathway (GO:0050857)	3.8116
T Cell migration (GO:0072678)	3.8250
Regulation of B cell receptor signaling pathway (GO:0050855)	3.6574
Leukocyte cell-cell adhesion (GO:0007159)	3.5362
Positive regulation of intracellular signal transduction (GO:1902533)	3.4337
Positive regulation of cytokine biosynthetic process (GO: 0042108)	3.3738
Negative regulation of lymphocyte proliferation (GO: 0050672)	3.3443
Positive regulation of MAPK cascade (GO: 0043410)	3.2727
Hematopoietic progenitor cell differentiation (GO: 0002244)	2.9904
Regulation of T cell differentiation (GO: 0045580)	2.9747
Negative regulation of cytokine production (GO: 0001818)	2.9568
Regulation of lymphocyte differentiation (GO: 0045619)	2.8904
Adaptive immune response based on somatic recombination of immune receptors built from immunoglobulin superfamily domains (GO: 0002460)	2.9004
Positive regulation of tumor necrosis factor production (GO: 0032760)	2.9087
Positive regulation of cytokine production (GO: 0001819)	2.7734
Regulation of interleukin-4 production (GO: 0032673)	2.7612
Positive regulation of interleukin-4 production (GO: 0032753)	2.7704
Positive regulation of leukocyte mediated cytotoxicity (GO: 0001912)	2.7287
T-helper cell lineage commitment (GO: 0002295)	2.7292
**2019 human KEGG pathways**	**−log10-*p* value**
Hematopoietic cell lineage	11.7752
T cell receptor signaling pathway	9.6088
Chemokine signaling pathway	8.8162
Cell adhesion molecules (CAMs)	7.9292
Cytokine-cytokine receptor interaction	7.7867
Primary immunodeficiency	7.6446
Natural killer cell mediated cytotoxicity	7.2240
Th17 cell differentiation	6.0255
Th1 and Th2 cell differentiation	5.1835
JAK-STAT signaling pathway	3.7669
Intestinal immune network for IgA production	3.4458
Graft-versus-host disease	3.0085
Antigen processing and presentation	2.7833

### Key Genes of the Co-expression Immune Network Do Not Strongly Correlate With FIGO Stage

**FIGURE 3:**
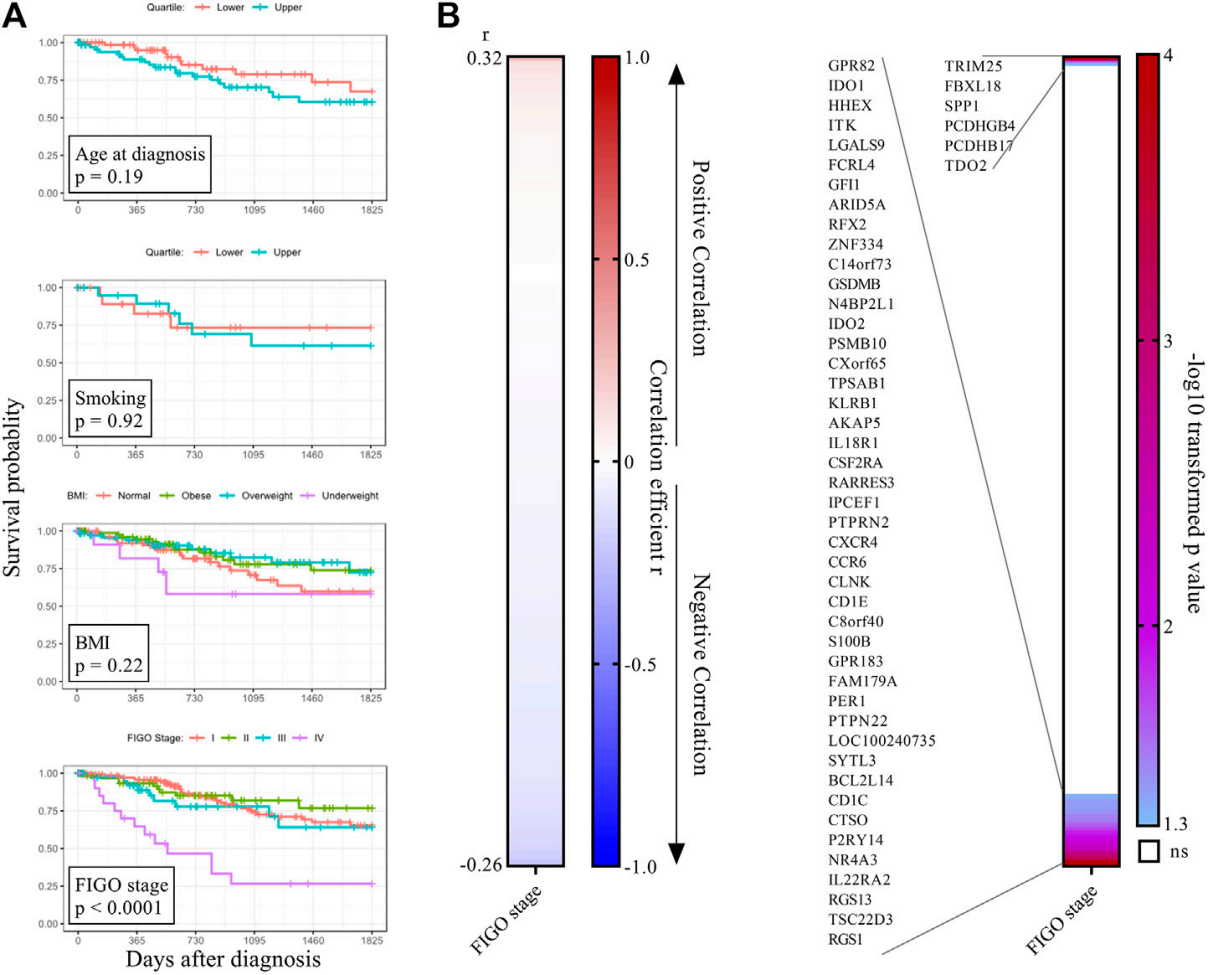
CESC FIGO stage is associated with tumour-promoting proinflammatory cytokines. **(A)** Clinical features were tested for association with survival. For BMI, four groups were defined based on diagnostic BMI values (underweight <18.5, normal 18.5–24.9, overweight 25–30, obese >30). For smoking, the lower and upper quartile of the clinical feature pack_years_smoked were compared. **(B)** Genes of the WGCNA palevioletred1 Immune Module (*n* = 505) which significantly associated with 5-years survival probability with cervical cancer were analyzed for correlation with FIGO stage using Spearman correlation analysis. Genes were ordered from largest to smallest correlation coefficient r (left panel). *p*-values were–log10 transformed and plotted as heatmap (right panel). Significant genes at the top are positively correlated with FIGO stage and significant genes at the bottom are negatively correlated with FIGO stage.

To test whether clinical features contributed to survival probability in the TGCA CESC cohort, we performed correlation analysis for age, body-mass-index, and use of tobacco products, and none proved predictive (Figure 3A). No difference in survival probability was observed for patients presenting with FIGO stages I, II, and III CESC, whereas FIGO stage IV presentation was as expected associated with decreased survival (Figure 3A). To test whether FIGO staging correlated with the expression of survival-associated immune genes, we performed a Spearman Correlation analysis for every of the 505 survival-associated genes of the WGCNA palevioletred2 Immune Module, identifying 51 significant FIGO-stage correlated genes, although with weak correlation (Figure 3B). 45 of these 51 genes had a weak negative correlation with FIGO stage, and were largely independent in a PPI analysis (not shown).

To identify survival-associated immune genes that associated with FIGO staging but were not part of the co-expression Immune Module, we further carried out a knowledge based analysis of immune genes from recognized KEGG immune pathways for association with survival. From these, we identified 220 genes significantly associated with 5-years survival probability, of which 155 were associated with higher survival probability when expressed at high level, while 65 were associated with lower survival probability when expressed at high level (Supplementary Figure S1A,B). Also, 108 of the 220 genes overlapped with the WGCNA palevioletred2 Immune Module. We tested FIGO stage correlation with these 220 survival-associated immune genes, and identified 27 significant FIGO-stage correlated genes (Supplementary Figure S2). Although not part of the WGCNA palevioletred2 Immune Module, we identified IL1A, IL1B and TGFB1 as positively correlated with FIGO stage, suggesting a contribution of inflammation to cervical cancer progression. The level of expression of MHC class II genes and IL12A was negatively correlated with FIGO stage, indicating that tumors with an increased FIGO stage might be depleted of antigen-presenting cells.

### Expression of Multiple Inhibitory Immune Checkpoint and Treg Related Genes is Associated With Increased 5-years Survival Probability With CESC

**FIGURE 4:**
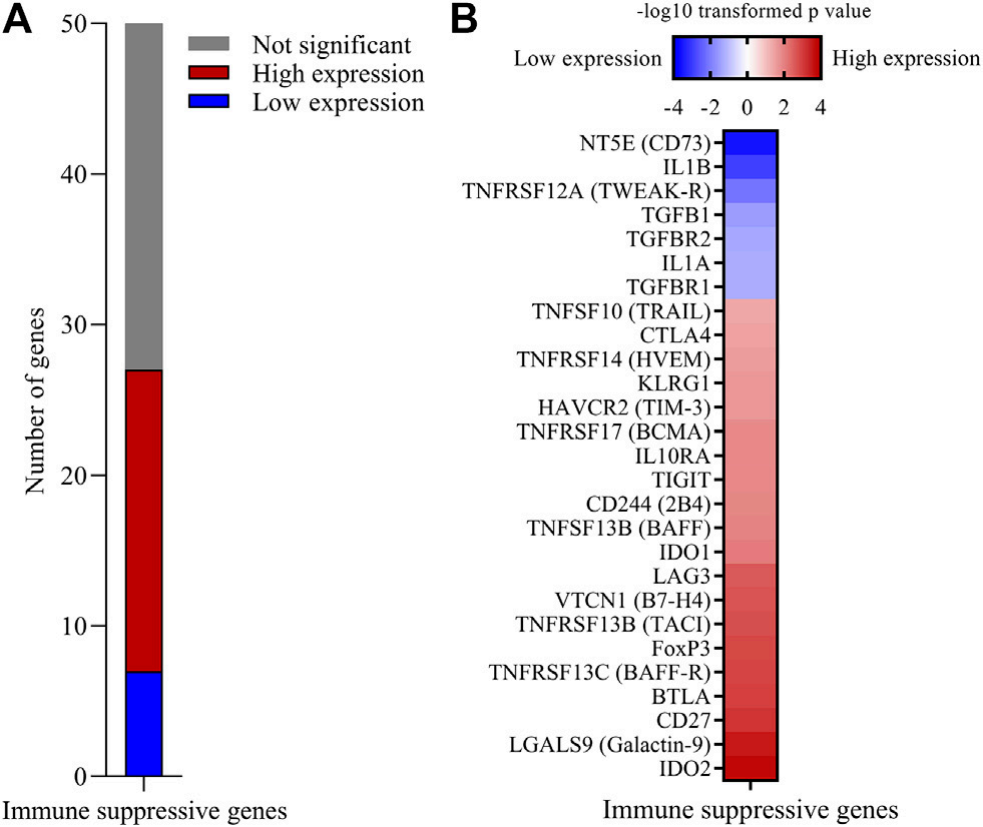
High expression of multiple immune suppression genes is associated with increased 5-years survival probability with CESC. A custom built list of 50 immune suppressive genes including immune checkpoint inhibitor genes, Treg related genes and soluble mediators was analyzed for significant association with 5-years survival probability. **(A)** Number of genes where high or low expression was associated with increased 5-years survival probability. **(B)** *p*-values of significant immune suppression genes were −log10 transformed. Genes for which low expression was associated with increased survival probability were further transformed to a negative value.

Progressing tumors express inhibitory immune molecules and/or regulatory T cells (Tregs). Inhibitory molecule targeting has revolutionized cancer immunotherapy, but less than 20% of cervical cancer patients are responsive to current immune checkpoint inhibition therapy (Sharma et al., 2017). We created a knowledge-based list of 50 immune-suppression associated genes (Figure 1A; (Supplementary File S1) including many inhibitory immune checkpoint genes, Treg associated genes and soluble immune-suppressive mediators, and found that high expression of 20 of these genes and low expression of seven was significantly associated with increased 5-years survival probability (Figure 4A). Genes where high expression associated with better survival encoded B7H4, Galactin-9, HVEM, TACI, BAFF, BAFF-R, CTLA4, LAG3, TIGIT, TRAIL, as well as Treg associated genes FOXP3, KLRG1, and IL10RA, and soluble mediators IDO1 and IDO2 (Figure 4B). In addition to classical tumor-promoting TGFB family and IL1 family genes, immune checkpoints CD73 (NT5E) and TWEAK-R (TNFRSF12A) were identified as immune suppressive genes for which high expression was associated with a decreased survival probability, suggesting that these molecules might be targets for immunotherapy for subjects with this CESC phenotype. The WGCNA co-expression network also contained 16 of the 20 survival-associated immune suppressive genes, including 11 immune checkpoint genes, Treg associated genes as well as IDO1 and IDO2 (Supplementary File S1).

### Overall Immune Activity is Highly and Positively Correlated With Immune Suppression, Predicting Survival Probability in CESC and HNSCC

**FIGURE 5:**
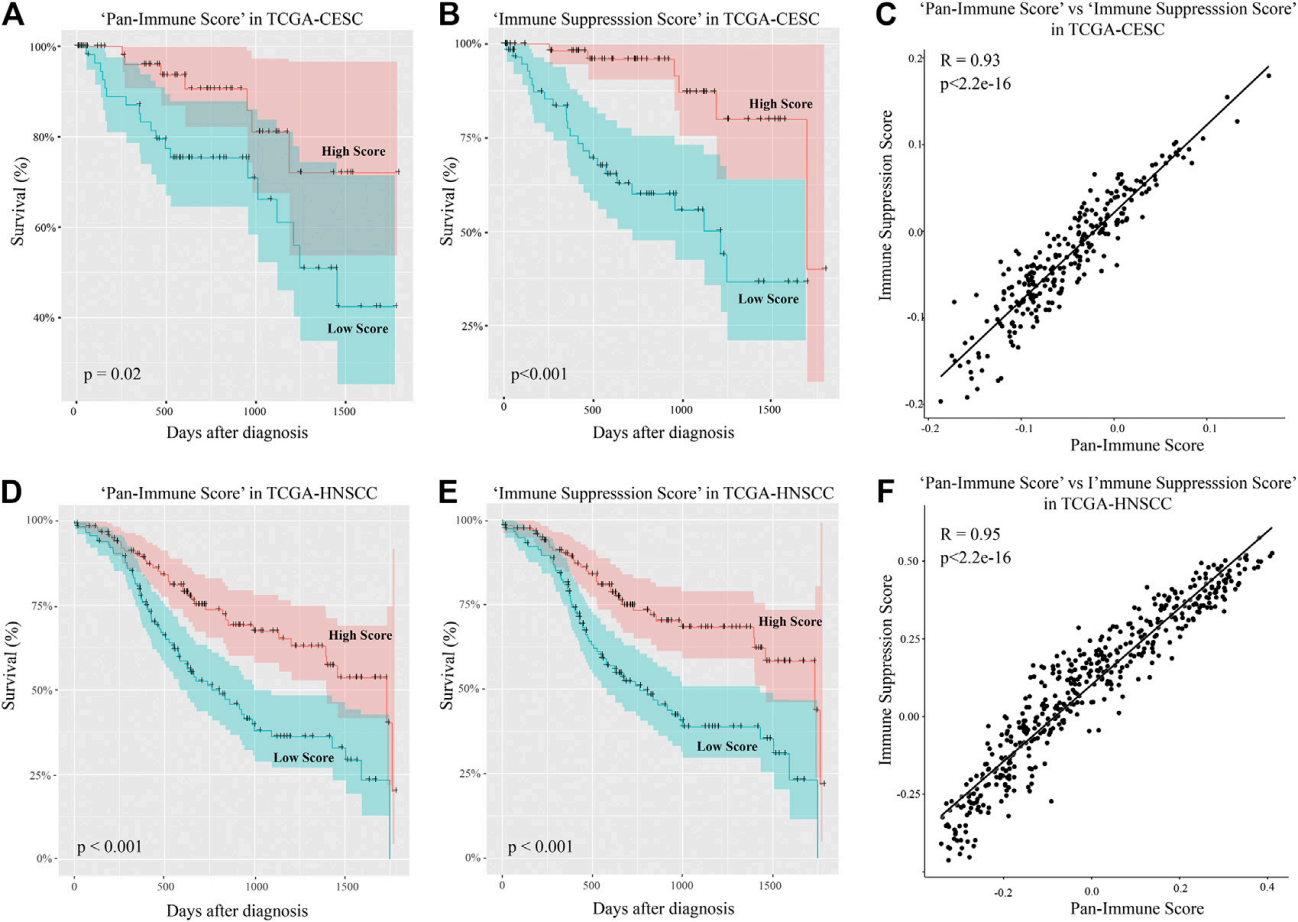
“Pan-Immune Score” and “Immune Suppression Score” predicting 5-years survival probability with CESC and HNSCC are highly correlated. **(A, D)** Subjects were assigned a “Pan-Immune Score” according to expression of survival-associated immune genes of the WGCNA palevioletred1 Immune Module, and 5-years survival probability of the upper and lower quartiles of the “Pan-Immune Score” representing high and low scores was compared in the TCGA-CESC **(A)** and TCGA-HNSCC **(D)** data. **(B, E)** Subjects were assigned an “Immune Suppression Score” according to expression of survival-associated immune suppression genes described in Figure 1B, and 5-years survival probability of the upper and lower quartiles of the “Immune Suppression Score” representing high and low scores was compared in the TCGA-CESC **(B)** and TCGA-HNSCC **(E)** data. **(C, F)** Correlation analysis of “Pan-Immune Score” and “Immune Suppression Score” in the TCGA-CESC **(C)** and TCGA-HNSCC **(F)** data.

To assess the relationship between immunity-promoting and immune-suppressive gene activity in CESC, we built scores using a method developed by Foroutan and colleagues (Foroutan et al., 2018). A “Immune Suppression Score” was constructed based on the previously identified 20 immune-suppressive genes described in Figure 4B, for which high expression was associated with increased survival probability (Supplementary File S1). An overall “Pan-Immune Score” was built based on the 462 survival-associated genes of the palevioletred1 Immune Module excluding any overlapping immune-suppressive genes present in the “Immune Suppression Score” to ensure both scores are independent (Supplementary File S1). We subsequently compared 5-years survival probability for subjects of the upper (high) and lower (low) score quartiles. CESC subjects with a high “Pan-Immune Score” had significantly increased 5-years survival probability compared to subjects with a low “Pan-Immune Score” (Figure 5A). Interestingly, CESC subjects with a high “Immune Suppression Score” also had significantly increased 5-years survival probability compared to subjects with a low “Immune Suppression Score” (Figure 5B). “Pan-Immune Score” and “Immune Suppression Score” were highly positively correlated in CESC (Figure 5C). We tested the applicability of the TCGA CESC-derived-“Pan-Immune Score” and “Immune Suppression Score” to data from the TCGA HNSCC cohort. As observed for CESC, a high “Pan-Immune Score” was associated with increased 5-years survival probability in HNSCC, indicating that survival probability in HNSCC is also linked to a hot/cold tumor phenotype (Figure 5D). A high “Immune Suppression Score” was also significantly associated with increased survival probability in HNSCC (Figure 5E), and as observed in the CESC cohort data, “Pan-Immune Score” and “Immune Suppression Score” were also highly positively correlated in HNSCC (Figure 5F). These data suggest that CESC and HNSCC patients with increased survival probability express higher levels of immune-suppressive genes as a consequence of overall immune activity within the tumor.

**FIGURE 6:**
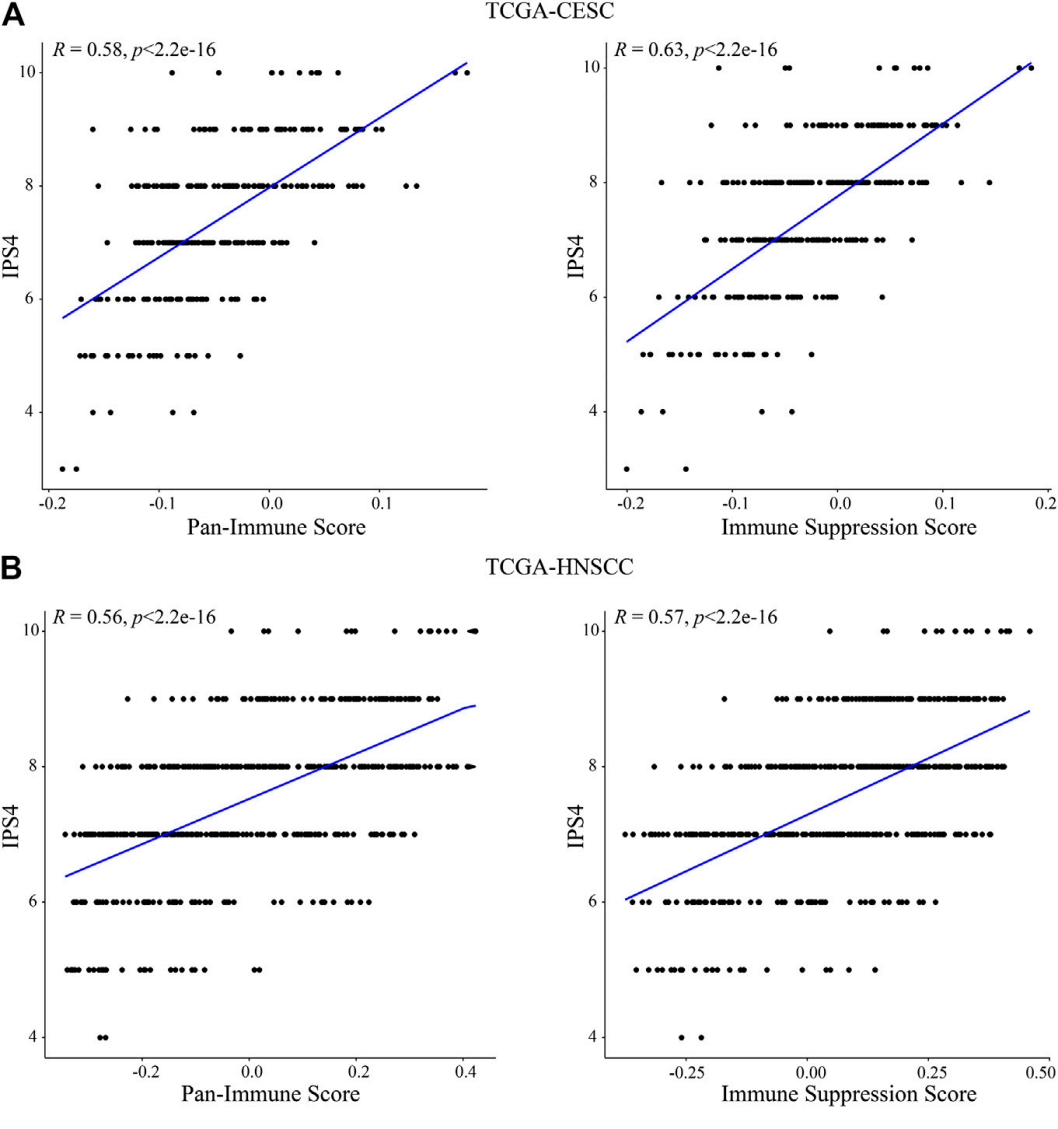
“Pan-Immune Score” and “Immune Suppression Score” are positively correlated with “Immunophenoscore.” Correlation analysis of “Pan-Immune Score” and “Immune Suppression Score” with “Immunophenoscore” IPS4 in TCGA-CESC **(A)** and TCGA-HNSCC **(B)**.

To compare these findings with previously established immune scores, we assessed correlation of the “Pan-Immune Score” and “Immune Suppression Score” with the “Immunophenoscores” IPS1-4 which have been shown to predict response to immune checkpoint inhibition therapy in melanoma patients (Charoentong et al., 2017). In both CESC and HNSCC, both “Pan-Immune Score” and “Immune Suppression Score” were positively and significantly correlated with IPS2-4, with the strongest correlation observed with IPS4 (Table 3; Figures 6A,B). IPS2-4, in contrast to IPS1, are positively weighted for expression of CTLA4 (IPS2), PD1/PDL1/PDL2 (IPS3) or CTLA4/PD1/PDL1/PDL2 (IPS4). The lack of positive or strong correlation of IPS1 with the here established scores is likely due to IPS1 weighing inhibitory immunomodulators negative, resulting in a lower score of samples expressing high levels of these molecules, while the “Immune Suppressive Score” and “Pan-Immune Score” weigh expression of any immune-related genes positively toward the score.

**TABLE 3:** Correlation of IPS1-4 with “Pan-Immune Score” and “Immune Suppression Score” in CESC and HNSCC. *R*: Correlation efficient; *p*: *p*-value.

	IPS1		IPS2		IPS3		IPS4	
	*R*	*p*	*R*	*p*	*R*	*p*	*R*	*p*
CESC								
Pan-immune score	0.0027	0.96	0.45	<2.2e-16	0.2	0.00056	0.58	<2.2e-16
Immune suppression score	0.15	0.011	0.54	<2.2e-16	0.32	7.9e-09	0.63	<2.2e−16
HNSCC								
Pan-immune score	−0.12	0.0065	0.42	<2.2e-16	0.13	0.0029	0.56	<2.2e-16
Immune suppression score	−0.063	0.15	0.43	<2.2e-16	0.19	1.9e-05	0.57	<2.2e-16

Combined with the observation that tumors presenting with an overall increased immune gene co-expression are likely to demonstrate an increased probability of 5-years survival, these data suggest that CESC and HNSCC tumors with high levels of expression of specific immune-suppressive genes are more associated with a high probability of 5-years survival than CESC and HNSCC tumors lacking any immune related gene expression.

## Discussion

The current consensus is that cancers of most organs and tissues are extensively heterogeneous, consistent with the observation that only a limited proportion of patients with any particular tumor type respond to any specific immunotherapy. Advancements in high-dimensional screening technologies have enabled consideration of personalized cancer immunotherapy, optimized for the phenotype of the patient’s tumor. We aimed in the current study to determine the co-expressed immune gene networks within CESC tumors that correlate with survival, and to analyze survival-associated immune genes which are related to immune suppression, in an attempt to subdivide primary CESC tumors by immune phenotype, which may guide appropriate immunotherapy selection.

Dominant co-expressed immune networks associated with increased survival probability were T cell related, as well as enriched in pathways of cytokine and chemokine signaling. Others have predicted the tumor-infiltrating immune cell composition in the TCGA CESC dataset using the metagene CIBERSORT approach, and found in line with our data that an enrichment of T cells was associated with increased survival probability (Wang et al., 2019). In addition to CD8 T cells, high infiltration of resting but not activated mast cells, stationary dendritic cells, and activated CD4 T cells in CESC were also found related to better survival (Yang et al., 2019; Nie et al., 2020). Resting mast cells and naïve B cells were recently selected as prognostic signatures (Che et al., 2020), where SCC patients were stratified into low and high risk groups. The low risk patients with high proportions of these two immune cells had better survival rates than high risk patients. Low risk patients significantly benefited from chemotherapeutic and immunotherapeutic treatments. In addition to tumor-infiltrating immune cells, immune-related gene sets such as RIPOR2, DAAM2, SORBS1, and CXCL8 (Mei et al., 2020), or LTA, TFRC, TYK2, DLL4, CSK, JUND, NFATC4, SBDS, FLT1, IL17RD, IL3RA, SDC1, PLAU (Ding et al., 2020), were proposed as prognostic genes to stratify CESC patients. However, neither of these studies took co-expression of immune networks into consideration.

Subjects with primary CESC tumors expressing the lowest quartile of gene expression of co-expressed immune networks, suggestive of a “cold” tumor phenotype, had the lowest survival probability. Additionally, subjects with the lowest survival probability expressed very few immune-related genes at high levels, many of which are known to promote tumor progression, including IL1A, IL1B, and TGFB, and which were also correlated with increased FIGO stage. In a previous study, using immune related gene pairs (IRGPs), 29 IRGPs (47 genes) were proposed as prognostic immune markers to stratify CESC patients into low and high risk groups, and IL1B was also correlated with CESC development in the current study (Nie et al., 2020).

The current paradigm separates tumors into “hot,” with a substantial immune and inflammatory cell infiltrate, “cold,” lacking such an infiltrate, and “altered” phenotypes (Galon and Bruni, 2019). The “altered” phenotypes can be further divided into “altered-excluded” and “altered-immunosuppressed.” The former describes tumors containing immune cells in the tumor margin, but not within the tumor bed, and the latter describes tumors containing an overall intermediate “Immunoscore” (defined by CD3 and CD8 immunohistochemistry) while also expressing immune-suppressive molecules such as TGFβ and inhibitory immune checkpoint genes. When analyzing the TCGA CESC data for association of immune suppression genes with survival, including inhibitory immune checkpoint genes, we observed that subjects with tumors expressing the lowest levels of multiple inhibitory immune checkpoint and other immune suppression genes had the lowest survival probability, indicating that the tumor phenotype stratification can be ordered into a hierarchy. Our data suggests that subjects with “cold” CESC tumors have worse survival probabilities compared to subjects with “immunosuppressed” CESC tumors, at least when considering expression of the particular immunosuppressive genes outlined in this study. Our data further suggests that an “altered-immunosuppressed” classification is oversimplified and requires more precise characterization based on the expression of specific immune-suppressive molecules. Subjects with the top 25% of “Immune Suppression Score” had the best survival probability in both CESC and HNSCC. Furthermore, the high correlation between the overall “Pan-Immune Score” and the “Immune Suppression Score” demonstrates that CESC and HNSCC tumors with the highest levels of immune activity also express the highest levels of particular inhibitory immune checkpoint genes and Treg associated genes, and despite this are associated paradoxically with the best survival probability following conventional therapy. We therefore hypothesize that a combined specific immune checkpoint inhibitor therapy and conventional therapy will likely further increase survival outcomes of CESC and HNSCC patients who already have high levels of immune gene expression within the tumor as indicator of tumor immune cell infiltration and priming of an anti-tumor T cell response, but might not benefit patients with “cold” tumors, as they lack an adaptive immune infiltrate, and testing this hypothesis should be an aim of future trials of immunotherapy.

Our analysis identified 20 inhibitory immune modulator genes for which high expression was associated with increased survival. In a recent report, eight inhibitory immune modulators in SCC were also identified as prognostic makers (Che et al., 2020), of which IDO1, TIGIT, ICOS, and LAG3 were consistent with our observation. We also identified seven genes, including TWEAK-R and CD73, for which high expression was associated with decreased survival, and we observed a comparable pattern in HNSCC. TWEAK-R is overexpressed in other multiple cancers and treatment with anti-TWEAK-R antibodies attracted effector immune cells infiltrating into tumors in mice (Ye et al., 2017). Targeting CD73 using antibodies or small molecule inhibitors has been proposed as immunotherapy to enhance T cell infiltration, and has been evaluated in different clinical settings, including cervical cancer (Arab and Hadjati, 2019). Hence, these targets represent promising therapy options for patients presenting with an overall “cold” tumor phenotype but expressing high levels of these molecules.

The most widely studied checkpoint inhibitors in cervical cancer target PD-1 and PD-L1, although response rates when given as monotherapy are below 20%. While neither PD-1 nor PD-L1 gene expression levels were associated with survival probability in the TCGA-CESC dataset, others have detected a significant correlation between expression of membrane PD-1 measured by immunohistochemistry with survival in patients with recurrent cervical cancer (Roszik et al., 2018). A recent study showed that high expression of PD-L1 on immune cells was associated with decreased survival, while high expression of PD-L1 on tumor cells was associated with increased survival (Chen et al., 2020), indicating another level of complexity of how these molecules effect outcomes. Lack of association of PD-1/PD-L1 gene expression at presentation with survival in this cohort has thus no implications for use of checkpoint inhibitors targeting these molecules as they may be induced by other therapies including HPV oncoprotein targeted vaccination.

Immunotherapy will need to be optimized to increase survival probability for patients presenting a “cold” tumor phenotype (Bonaventura et al., 2019). HPV-induced cervical cancers can be immunologically “cold” despite expressing virus derived antigens, implying that a cold phenotype can also arise from defects in antigen presentation. In this study we observed that the expression of MHC class II genes was negatively correlated to FIGO stage, indicating that antigen presentation can decline with tumor progression. Absence of MHC class I gene expression by tumors could also be associated with a “cold” tumor phenotype, but we did not identify any survival associated MHC class I genes in this data set. We speculate that patients with “cold” CESC and HNSCC tumors might benefit from a multi-step therapy approach (Frazer and Chandra, 2019) by: 1) conversion of “cold” to “hot” tumors using local radiation therapy to induce immunogenic cell death, and/or intra-tumor application of adjuvant (if achievable), 2) priming of a systemic tumor-targeted T cell response using HPV oncoprotein targeted vaccination for HPV + CESC and HNSCC (Chandra et al., 2017), 3) immune checkpoint inhibition to prolong tumor- and therapy-induced immune responses, 4) promotion of T cell homing to the tumor by blockade of TGFβ (Mariathasan et al., 2018), 5) anti-angiogenesis therapy by VEGF blockage, and 6) inhibition of tumor cell dissemination by targeting the central focal adhesion pathway (Budhwani et al., 2020). Proof of principle studies have shown that “cold” to “hot” tumor conversion can be achieved, for example by intra-tumor delivery of oncolytic viruses such as Talimogene laherparpvec (T-VEC), an engineered herpes simplex virus which selectively infects cancer cells and expresses the dendritic cell attractant granulocyte-macrophage colony-stimulating factor (GM-CSF) (Johnson et al., 2015). T-VEC is currently approved for use in non-operable melanoma lesions, with an overall response rate when given as monotherapy of 26.4% (Andtbacka et al., 2015). However, combination with PD-1 checkpoint blockade increased the overall response rate to 62% (Ribas et al., 2017), underlining the potential that lies in well-orchestrated combination immunotherapies. Targeting of the autophagy-related protein PIK3C3 (Vps34) has recently been demonstrated as a novel strategy to fire up “cold” tumors (Noman et al., 2020). A multi-faceted trial combining four of the above outlined approaches is currently underway in patients with checkpoint naïve advanced HPV-associated malignancies (NTC04287868). This study is testing a combination of a therapeutic HPV vaccine together with tumor-targeted IL-12 as “cold” to “hot” tumor converter (Strauss et al., 2019), and a bi-functional fusion protein blocking PD-L1 and TGFβ (Strauss et al., 2018) which has demonstrated enhanced efficacy in preclinical models of HPV-associated disease (Rumfield et al., 2020). Combination therapies may deliver better outcomes, but will also likely increase the risk of adverse and off target effects.

The data presented here and by others supports the hypothesis that the magnitude of a T cell-associated immune signature is predictive of prognosis in CESC and HNSCC. Our future outlook predicts that cancer patients will eventually have access to high-dimensional technologies to identify their individual tumor/immune landscape in detail and allow best suited drug selection from an extensive cancer- and immunotherapy drug palette.

## Data Availability

The original contributions presented in the study are included in the article/Supplementary Material, further inquiries can be directed to the corresponding author.
